# A novel hydroxycinnamoyl transferase for synthesis of hydroxycinnamoyl spermine conjugates in plants

**DOI:** 10.1186/s12870-019-1846-3

**Published:** 2019-06-17

**Authors:** Hui Peng, Rachel S. Meyer, Tianbao Yang, Bruce D. Whitaker, Frances Trouth, Lingfei Shangguan, Jingbing Huang, Amy Litt, Damon P. Little, Hengming Ke, Wayne M. Jurick

**Affiliations:** 1 Food Quality Laboratory, Beltsville Agricultural Research Center, Agricultural Research Service of U.S. Department of Agriculture, Beltsville, MD 20705 USA; 2 The Genome Center and Department of Plant Sciences, University of California, Davis, CA 95616 USA; 3 Department of Ecology and Evolutionary Biology, University of California, Los Angeles, Los Angeles, CA 90095 USA; 4 College of Horticulture, Nanjing Agricultural University, Nanjing, 210095 Jiangsu China; 5 College of Food Science and Engineering, Huazhong Agricultural University, Wuhan, 430070 Hubei China; 6 College of Natural and Agricultural Sciences, University of California, Riverside, CA 92521 USA; 7 Cullman Program for Molecular Systematics, New York Botanical Garden, 2900 Southern Boulevard, Bronx, New York, NY 10458 USA; 8 Department of Biochemistry and Biophysics, University of North Carolina at Chapel Hill, Chapel Hill, NC 27599 USA

**Keywords:** Eggplant, Hydroxycinnamic acid amide, Spermine hydroxycinnamoyl transferase, Substrate specificity, Crop improvement, *Solanum richardii*, Phytochemicals

## Abstract

**Background:**

Hydroxycinnamoyl-spermine conjugates (HCSpm) are a class of hydroxycinnamic acid amides (HCAAs), which not only are instrumental in plant development and stress response, but also benefit human health. However, HCSpm are not commonly produced in plants, and the mechanism of their biosynthesis remains unclear. In previous investigations of phenolics in *Solanum* fruits related to eggplant (*Solanum melongena* L.), we discovered that *Solanum richardii*, an African wild relative of eggplant, was rich in HCSpms in fruits.

**Results:**

The putative spermine hydroxycinnamoyl transferase (HT) *SpmHT* was isolated from *S. richardii* and eggplant. *SrSpmHT* expression was high in flowers and fruit, and was associated with HCSpm accumulation in *S. richardii*; however, *SpmHT* was hardly detected in eggplant cultivars and other wild relatives. Recombinant SpmHT exclusively selected spermine as the acyl acceptor substrate, while showing donor substrate preference in the following order: caffeoyl-CoA, feruloyl-CoA, and *p*-coumaroyl-CoA. Molecular docking revealed that substrate binding pockets of SpmHT could properly accommodate spermine but not the shorter, more common spermidine.

**Conclusion:**

SrSpmHT is a novel spermine hydroxycinnamoyl transferase that uses Spm exclusively as the acyl acceptor substrate to produce HCSpms. Our findings shed light on the HCSpm biosynthetic pathway that may allow an increase of health beneficial metabolites in *Solanum* crops via methods such as introgression or engineering HCAA metabolism.

**Electronic supplementary material:**

The online version of this article (10.1186/s12870-019-1846-3) contains supplementary material, which is available to authorized users.

## Background

Hydroxycinnamic acid amides (HCAAs) are a group of plant secondary metabolites found in a wide range of plant species [1–3]. Many studies have identified critical roles that HCAAs play in plant growth and developmental processes, including cell division, cytomorphogenesis, flowering, cell wall cross-linking, tuberization, and stress responses [2, 4, 5]. These compounds are antioxidants and effective free radical scavengers with anticarcinogenic, antihypertensive, antimicrobial, and other potentially therapeutic activity of significant benefit to human and animal health [6–8]. Due to the diversity of carbon skeletons, HCAAs can be divided into many categories such as the polyamine conjugates hydroxycinnamoyl-spermidine (HCSpd), −spermine (HCSpm), and -putrescine (HCPut). HCSpd and HCPut are predominant in the plant kingdom, while only a few plants are rich in HCSpm compounds [3, 9–11].

HCSpm, rare in nature, exhibit unique health benefits. For instance, N^1^, N^14^-bis (dihydrocaffeoyl) spermine (kukoamine A) is a major compound that confers the hypotensive and antiparasitic activities in fruit of the Chinese medicinal species *Lycium chinense* (goji berry) [3, 12–14]. Trypanothione reductase, an essential enzyme for survival of pathogenic protozoa such as Leishmania and other trypanosomes, is inhibited by a fourfold lower concentration of Kukoamine A compared with its spermidine counterpart [N^1^, N^10^-*bis* (dihydrocaffeoyl)-spermidine]. Kukoamine A also shows anticancer activity and attenuates insulin resistance and fatty liver disease [12, 14–17]. In addition, N^1^-coumaroylspermine, but not N^1^-coumaroylspermidine, is found to efficiently inhibit mammalian and crayfish neuroreceptors in vitro [18], an ability of great interest for pest management as well as pain management.

**Fig. 1:**
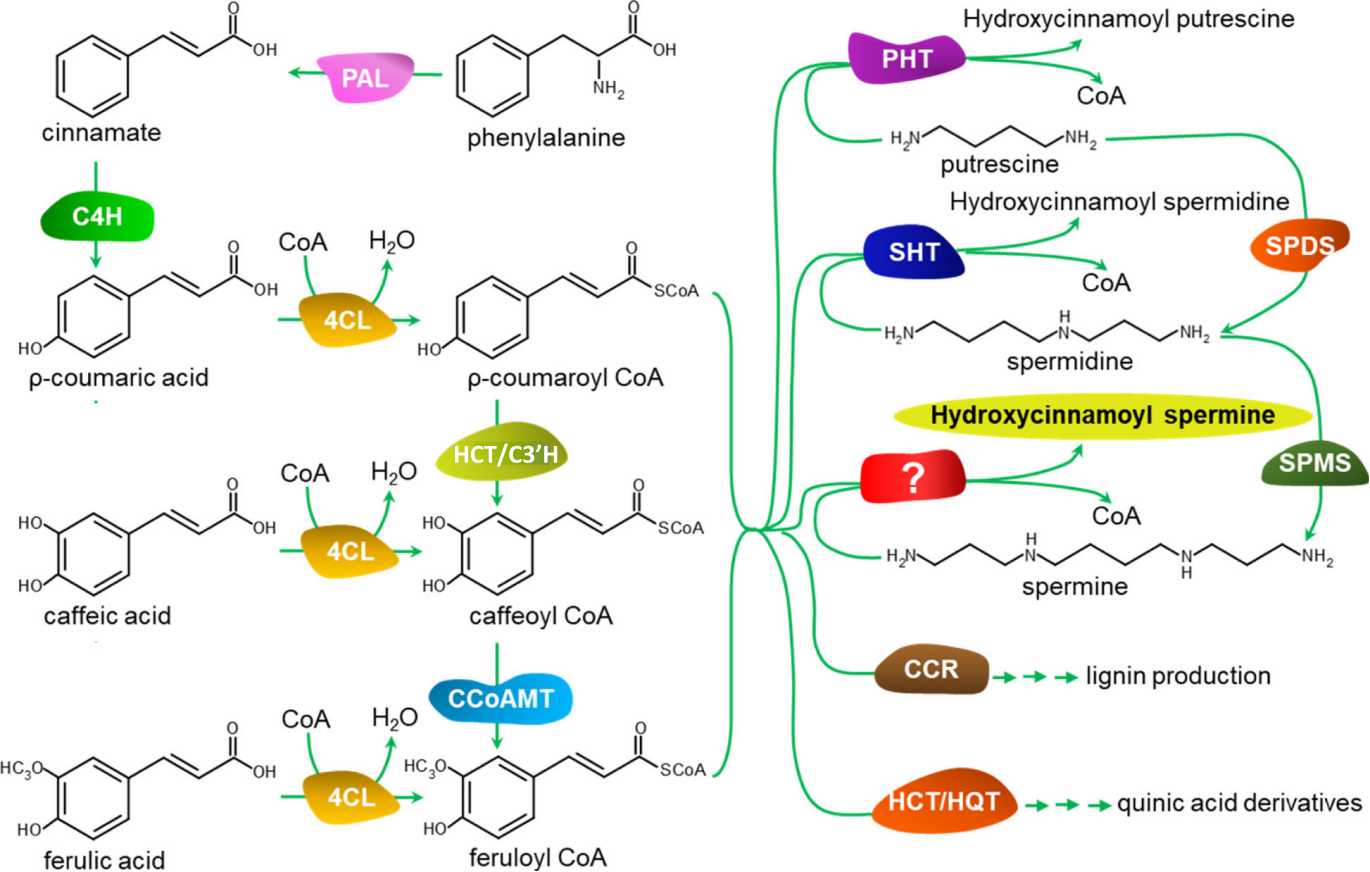
HCAA biosynthesis pathway in plants. PAL, phenylalanine ammonia lyase; C4H, cinnamate 4-hydroxylase; C3’H, cinnamate 3′-hydrolase; 4CL, 4-coumaroyl-coenzyme A ligase; CCoAMT, caffeoyl-CoA o-methyltransferase; CCR, cinnamoyl Co-A reductase; HCT, Hydroxycinnamoyl transferase; HQT, Hydroxycinnamoyl acid shikimate/quinate transferase; PHT, putrescine hydroxycinnamoyl transferase; SHT, spermidine hydroxycinnamoyl transferase; SPDS, spermidine synthase; SPMS, spermine synthase

In plants, HCAA compounds are synthesized via the phenylpropanoid pathway, which deploys numerous enzymes to convert phenylalanine into *trans*-cinnamate, hydroxycinnamoyl CoA thioesters [19, 20]. Hydroxycinnamoyl-CoAs diverted from synthesis of fruit-prevalent HCA conjugates can be condensed with polyamines by hydroxycinnamoyl transferases (HTs) to yield HCAAs (Fig. 1) [2, 21–24]. The first characterized HT was an *Arabidopsis thaliana* spermidine HT (AtSHT) that catalyzed the formation of mono-, di- and triacylated-spermidine [24]. Later, two more Arabidopsis SHT genes (AtSDT and AtSCT) were found to regulate the accumulation of disinapoyl-spermidine and dicoumaroyl-spermidine, respectively [2]. *Nicotiana attenuata* SHT (NaDH29) and putrescine HT (NaAT1) were able to catalyze the synthesis of HCSpd and HCPut, respectively [25]. Previously we identified an eggplant SHT (SmSHT) that was predicted to play a major role in the formation of HCSpd in flowers and fruit [26]. However, an HT specifically responsible for the biosynthesis of HCSpm has yet to be identified.

HTs belong to the BAHD superfamily in plants, which are acyl-CoA dependent acyltransferases [27–29]. Members of this superfamily are involved in synthesis or modification of diverse metabolites such as alkaloids, terpenoids and phenolics [28]. They possess a conserved catalytic motif (HXXXD) and a potentially conformation-related motif (DFGWG) [28, 30]. To date, crystal structures of a few BAHD proteins have been resolved, including a *Coffea canephora* shikimate hydroxycinnamoyl transferase (CcHCT) [31] and a *Sorghum bicolor* hydroxycinnamoyl transferase (SbHCT) [32]. These studies have partially elucidated the mechanisms of catalysis and substrate selection by BAHD enzymes.

The Nightshade family (*Solanaceae*), which includes *Lycium chinense* and *Nicotiana*, encompasses dozens of edible fruit crops with diverse HCA conjugates, among them HCAAs [33]. An exemplar nightshade crop is eggplant, a commercially important crop worldwide with great medicinal importance [34–36] that exceeds most vegetables in superoxide scavenging activity [37]. Previous work screening eggplant (*Solanum melongena* L.) and wild relatives from Africa, Asia, and South America have identified HCAA compounds using HPLC and LC-MS-TOF [33, 38–40]. Several wild relatives of eggplant shown to possess higher levels of HCA conjugates are currently being used in eggplant improvement breeding programs [41, 42]. These health-related qualities are largely attributable to abundant and diverse HCA constituents including HCAAs [38, 43, 44], the concentrations of which unfortunately have been lowered in the process of domestication [40].

Our previous investigations discovered a few accessions such as PI183357A (*Solanum insanum*), MM1506B (*Solanum insanum*), W324 (*Solanum macrocarpon*), and PI500922 (*Solanum richardii*) that produce HCSpms by screening 93 accessions in *Solanum* family [40]. Among of them, *Solanum richardii* is an African species that exhibits a strikingly different HCAA profile indicating the presence in fruit of extraordinary levels of unique HCSpm compounds [19, 33, 39, 40]. Correlation of these phytochemical profiles with the expression of several genes in their biosynthetic pathway was used to hypothesize the route of HCAA biosynthesis, suggesting additional candidate HT [45]. In this study, we analyzed the eggplant expression sequence tag (EST) databases, and identified a candidate SpmHT gene based on enzyme kinetics, structural modeling, and gene expression profiles in both eggplant and *S. richardii*.

## Results

### Identification of a putative spermine hydroxycinnamoyl transferase

**Fig. 2:**
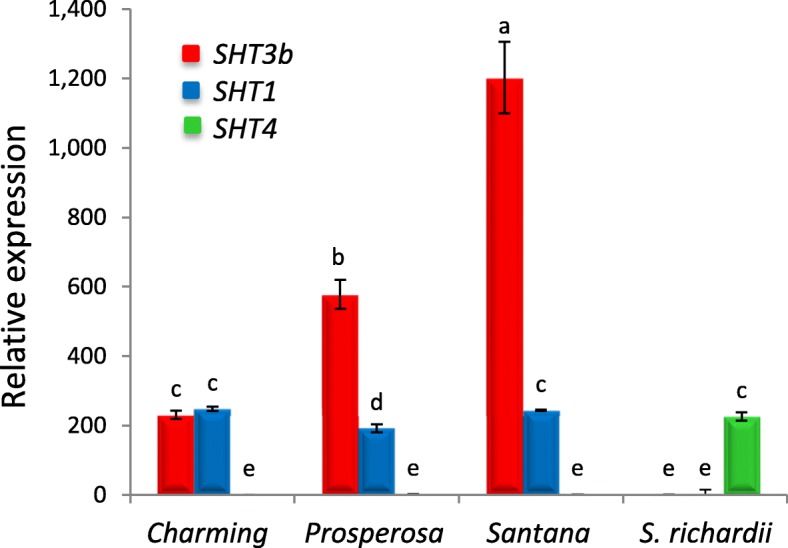
Expression of three putative polyamine hydroxycinnamoyl transferase genes in mature fruit of *S. melongena* (cv*.* Charming, Prosperosa, and Santana) and *S. richardii*. qRT-PCR was performed in triplicates using *GAPDH* as a constitutive control. Note that *SHT1* corresponded to *SHT* previously reported [26], while *SHT4* was renamed after *SpmHT* later. Relative expression was shown in fold changes (lowest value = 1). The data represent the mean value (± SD) of three biological replicates. Different letters indicate significant differences among mean values (*P* < 0.05; Tukey HSD test)

The fact that HCA-spermine conjugates are predominantly present in *S. richardii* suggests that a unique SpmHT is highly expressed in *S. richardii*, but not in eggplant and many wild species examined [40]. Since both eggplant and *S. richardii* genomes were not available in the beginning of this study, we searched eggplant EST database to find the SpmHT candidate against the coding sequences (CDSs) of *AtSHT*. Obtained ESTs were assembled into six contigs, designated as *SHT1* (ESTs: FS029005, FS008363, FS012273, FS020813, and FS067971), *SHT2* (FS075455 and FS075454), *SHT3b* (FS088019, FS032510, and FS088020), *SHT4* (FS076011, FS071986, and FS083413), *SHT4b* (FS078688), and *SHT5* (FS066003, FS041561, and FS034051). Three of them (*SHT2*, *SHT4b*, and *SHT5*) were not detected in fruit from both eggplant and *S. richardii* and thus were not pursued further. Two (*SHT1* and *SHT3b*) of other three genes were highly expressed in the mature fruit of all eggplant cultivars examined but barely in that of *S. richardii* (Fig. 2). Note that *SHT1* is reported by us previously [26]. However, *SHT4* was detected predominantly in fruit from *S. richardii*, and hardly detected in eggplant fruit, strongly suggesting that it is a *SpmHT* candidate. Hence we renamed *SHT4* after *SpmHT*.

**Fig. 3:**
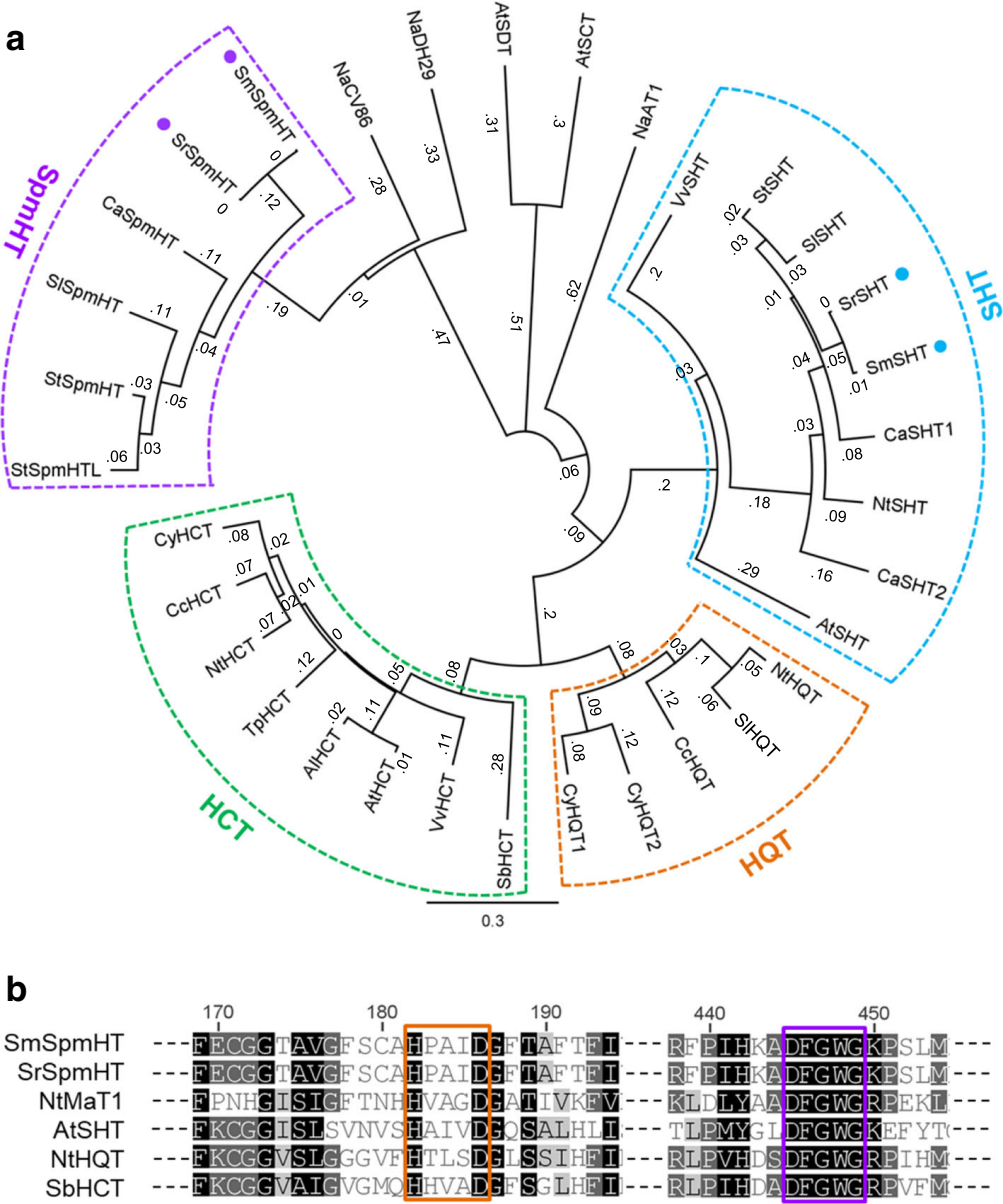
Phylogram of amino acid sequences of SpmHTs and other BAHD proteins. **a**, Phylogenetic tree constructed with SpmHTs and other BAHD proteins. Eggplant SHTs and SpmHTs are marked by solid blue and purple dots, respectively. The phylogram was generated by the neighbor-joining method following alignment by the ClustalW algorithm using Geneious software (version 4.8.5). The values represented the genetic distance under the Jukes-Cantor model. *Arabidopsis lyrata* AlHCT (EFH70827), *Arabidopsis thaliana* AtHCT (AT5G48930), AtSCT (AT2G25150), AtSDT (AT2G23510), AtSHT (AT2G19070), *Capsicum annuum* CaSHT1 (Capana00g001430), CaSHT2 (Capana05g000927), CaSpmHT (CA09g07820), *Coffea canephora* CcHCT (EF137954), CcHQT (EF153931), *Cynara cardunculus* CyHCT (DQ104740), CyHQT1 (EU697935), CyHQT2 (EU839580), *Lycopersicon esculentum* LsHQT (AJ582652), *Nicotiana attenuata* NaDH29 (JN390824), NaCV86 (JN390825), NaAT1 (JN390826), *Nicotiana tabacum* NtSHT (unannotated), NtHQT (AJ582651), NtHCT (CAD47830), *Sorghum bicolor* SbHCT (XP_002452435), *Solanum lycopersicum* SlSHT (Solyc07g015960.1.1), SlSpmHT (Solyc12g010980.1.1), *Solanum melongena* SmSHT (KP165410), SmSpmHT (KP233218), *Solanum richardii* SrSHT (KP165411), SrSpmHT (KR150683), *Solanum tuberosum* StSHT (PGSC0003DMP400059459), StSpmHT (Sotub12g006790.1.1), StSpmHTL (Sotub12g006750.1.1), *Trifolium pratense* TpHCT (EU861218), *Vitis vinifera* VvHCT (XP_002268988), and VvSHT (XP_002269790). **b**, Alignment of conserved regions of eggplant SpmHT and other polyamine hydroxycinnamoyl transferases. The conserved motifs ‘HXXXD’ and ‘DFGWG’ are marked by an orange and purple box, respectively

We cloned and sequenced the entire coding regions of *SrSpmHT* (KR150683) from *S. richardii* and its ortholog *SmSpmHT* (KP233218) from *S. melongena*. Sequence alignment revealed that both *SrSpmHT* and *SmSpmHT* encode putative proteins of 447 amino acids with only two amino acid substitutions. Among the functionally characterized acyltransferases from *Arabidopsis*, *Coffea*, *Nicotiana*, and *Solanum*, a tobacco SHT (NaCV86) showed the highest amino acid identity (53.6%) to both *Solanum* SpmHTs. Gene tree analysis revealed that SpmHT and other hydroxycinnamoyl transferases were largely in four clades (Fig. 3a). All functionally characterized shikimate hydroxycinnamoyl transferases (HCT) and quinate hydroxycinnamoyl transferases (HQT) fell into the clades we designated HCT and HQT, respectively. The clade containing Arabidopsis and eggplant SHT was designated SHT because both members have been confirmed to catalyze spermidine acylation [24, 26]. Two *Solanum* putative SpmHT formed a clade. Like other BAHD family proteins, they contained typical HXXXD and DFGWG motifs as well as some conserved catalysis-related sites (Fig. 3b and Additional file 1: Figure S1). However, the similarity in other regions was low compared with the BAHD proteins in other clades.

### SpmHT is a spermine-exclusive hydroxycinnamoyl transferase

**Fig. 4:**
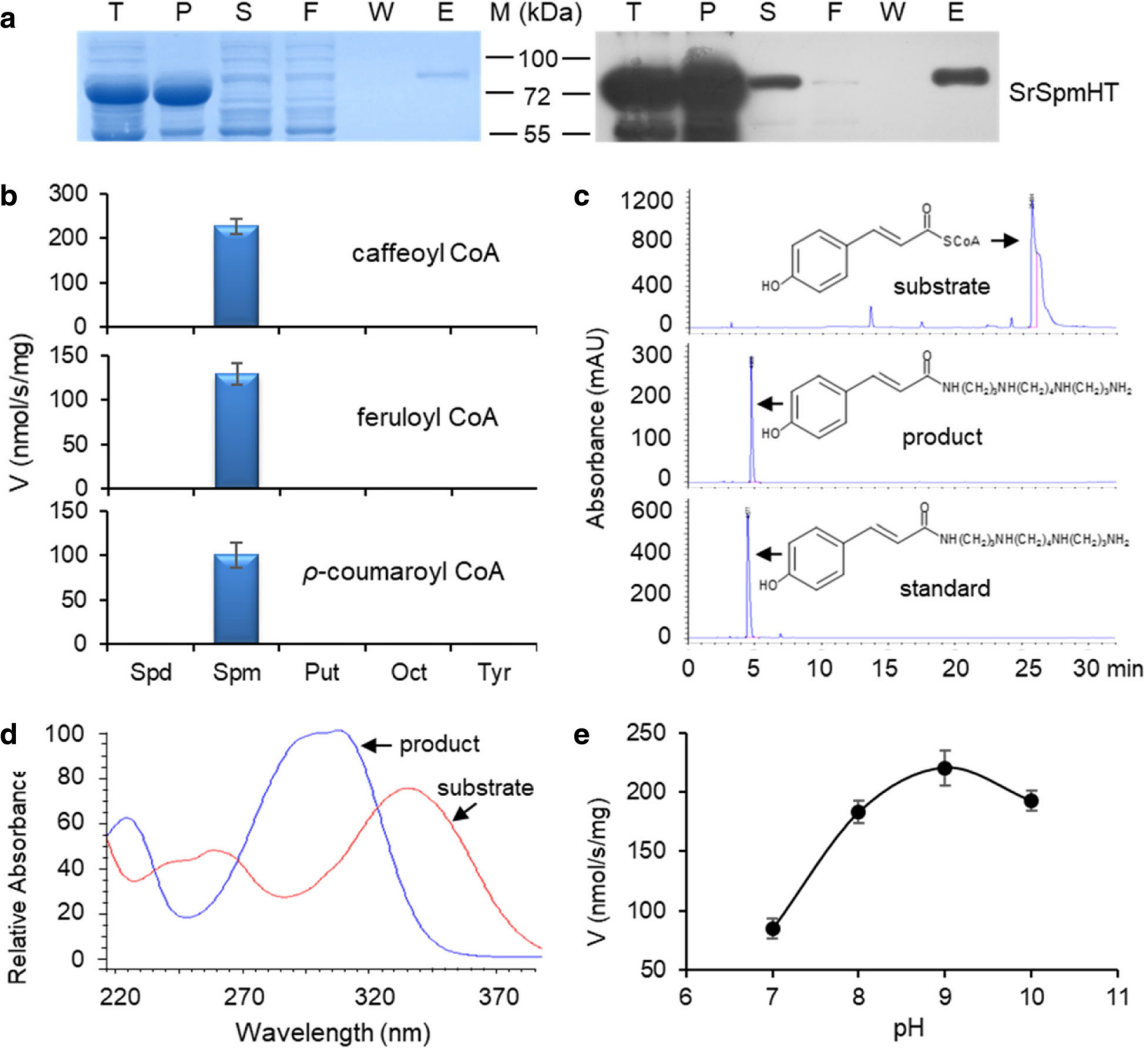
Substrate specificity and pH optima of SrSpmHT. **a**, Isolation of recombinant *S. richardii* spermine hydroxycinnamoyl tansferase (SrSpmHT). His-tagged recombinant proteins were expressed in *E. coli* and purified from cell lysates by nickel affinity chromatography. Evaluation of protein purity was performed by SDS-PAGE with coomassie blue staining of the gel (left), and Western blotting using anti-His-tag antibody (right): T, total cell lysate; P, cell pellet; S, supernatant; F, Flowthrough solution; W, last column wash; E, column eluates; **b**, Acyl donor substrate specificity of SrSpmHT. The activities were measured at 120 μM hydroxycinnaminoyl CoA (caffeoyl CoA, feruloyl-CoA and ρ-coumaroyl CoA) and 2.5 mM polyamine (spermidine, Spd; spermine, Spm; putrescine, Put, octopamine, Oct; tyramine, Tyr); **c**, C18-HPLC-DAD chromatogram of reaction products of SrSpmHT with spermine plus ρ-coumaroyl CoA as substrates (mAU, milli-absorption units). N1-ρ-coumaroyl-Spm was used as a standard; **d**, UV absorbance spectra of ρ-coumaroyl CoA (substrate) and N1-ρ-coumaroyl-Spm (product); **e**, pH-dependent activities of SrSpmHT. Reactions were performed with 60 μM caffeoyl CoA and 2.5 mM spermine at different pH environments. Bars show standard errors calculated from triplicates

**Fig. 5:**
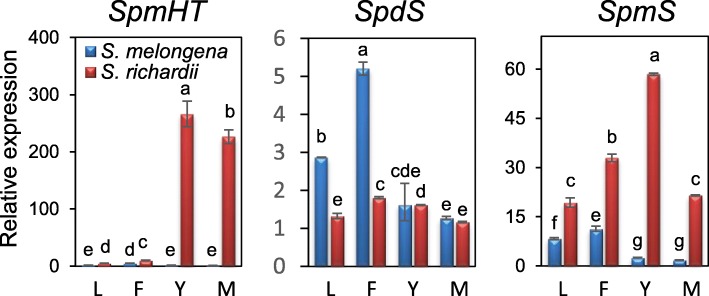
Expression patterns of *SpmHT*, *SpdS*, and *SpmS* in tissues of *S. melongena* and *S. richardii*. RNA was extracted from different tissues of mature plants, including leaves (L), flowers (F), young (Y) and mature (M) fruits. qRT-PCR was performed in triplicates with *GAPDH* as a constitutive control. Relative expression was shown in fold changes (lowest value = 1). The data represent the mean value (± standard deviation) of three biological replicates. Different letters indicate significant differences among mean values (*P* < 0.05; Tukey HSD test)

To investigate the catalytic activity of this hydroxycinnamoyl transferase, His-tagged recombinant SrSpmHT was purified from *E. coli* cell extracts and confirmed by both Coomassie Blue stained SDS-PAGE gel and Western blotting analyses with the anti-His antibody (Fig. 4a). Activity of SrSpmHT was tested with three acyl donor substrates (caffeoyl-, feruloyl- and *p*-coumaroyl-CoA) and five acyl acceptor substrates (Spd, Spm, Put, Oct, and Tyr). Spectrophotometric analysis showed that all tested HCA-CoA donor substrates could be efficiently utilized by SrSpmHT, whereas spermine was the only effective acceptor substrate (Fig. 4b). All reaction products were further examined by HPLC-DAD. As shown in Fig. 5c, only one peak was observed in the chromatogram when *p*-coumaroyl CoA and Spm were supplied as substrates. This peak was identified as N^1^-*p*-coumaroylspermine and gave the characteristic UV absorbance maximum at 322 nm (Fig. 4d). Similarly, caffeoyl-CoA and feruroyl-CoA also could be used as donor substrates by SrSpmHT to yield the corresponding monoacylated Spms. Further tests at different pH values revealed that SrSpmHT showed the highest activity at pH 9.0 (Fig. 4e). These results indicate that SpmHT is a spermine-exclusive hydroxycinnamoyl transferase.

Next, steady-state kinetics was investigated by varying acyl donor substrates. Before conducting the kinetics assay, we optimized the catalytic conditions to SrSpmHT including temperature (25, 30 and 37 °C), reaction time (5, 8, 11, 15 and 20 min), and pH (7.0 to 10.0) as well as the addition of DTT, EDTA, and Mg^2+^. The initial velocity was measured with different concentrations of the substrates at several reaction time points. SrSpmHT had the highest activity at pH 9.0 (Fig. 4e) and at 30 °C. The initial velocity was a straight line during 5–15 min. EDTA (10 mM) and Mg^2+^ (2 mM) had no detectable effect on the enzyme activity, whereas DTT (1 mM) clearly reduced activity (Additional file 2: Figure S2). We thus selected the optimized condition as 30 °C for 15 min with 10 mM EDTA at pH 9.0. The resulting plot line for hydroxycinnamoyl CoAs perfectly fit the Michaelis-Menten curve (R^2^ > 0.99, Additional file 3: Figure S3). The turnover number (*K*_cat_) of SrSpmHT for caffeoyl-CoA, feruloyl-CoA, and *p*-coumaroyl-CoA were 71.5, 13.9, and 8.5 s^− 1^, respectively, indicating that SrSpmHT prefers to use caffeoyl-CoA, followed by feruloyl-CoA and *p*-coumaroyl-CoA (Table 1). As for the catalytic efficiency (*K*_cat_/*K*_m_) of SrSpmHT, the calculated value for *p*-coumaroyl-CoA (0.173 s^− 1^ μM^− 1^) was significantly higher than those for caffeoyl-CoA (0.150 s^− 1^ μM^− 1^) and feruloyl-CoA (0.132 s^− 1^ μM^− 1^). In addition, eggplant SpmHT (SmSpmHT) was also expressed and purified from bacterial cells. The recombinant proteins showed the same substrate specificity, pH preference, and catalytic activity as SrSpmHT (Additional file 4: Figure S4), indicating that the two amino acid substitutions in these orthologs are not critical to the biochemical properties of the enzyme.

**Table 1:** Kinetic parameters of SrSpmHT with fixed concentrations of spermine and varying acyl donor substrates

Acyl donor	*V*_max_ (nmol•s^−1^•mg^− 1^)	*K*_m_ (μM)	*k*_cat_ (s^− 1^)	*k*_cat_/*K*_m_ (s^− 1^ μM^− 1^)
caffeoyl-CoA	1146.1 ± 101.4^a^	478.4 ± 46.5^a^	71.5 ± 6.3^a^	0.150 ± 0.001^b^
feruloyl-CoA	223.0 ± 8.0^b^	106.1 ± 9.0^b^	13.9 ± 0.5^b^	0.132 ± 0.006^c^
*ρ*-coumaroyl-CoA	135.8 ± 3.4^c^	49.0 ± 1.2^c^	8.5 ± 0.21^c^	0.173 ± 0.001^a^

Note: Different letters indicate significant difference on values (*P* < 0.05)

### *SpmHT* and *SpmS* are highly expressed in fruit from *S. richardii* but not eggplant

Expression patterns of *SpmHT* were examined in different tissues, including leaves, open flowers, young fruits, and mature fruits from eggplant (cv. Black Beauty) and *S. richardii*. *SrSpmHT* showed extremely high expression in both young and mature fruit of *S. richardii*. However, *SmSpmHT* was only weakly expressed in all examined eggplant tissues (Fig. 5). Previously we showed that *SHT* exhibited considerably lower expression in both young and mature fruits of *S. richardii* than those of eggplant [26]. These observations led us to hypothesize that the almost mutually exclusive expression of these genes in the two species could result in spermine being the predominant conjugated polyamine in *S. richardii*, whereas spermidine is the main conjugated polyamine in eggplant.

**Fig. 6:**
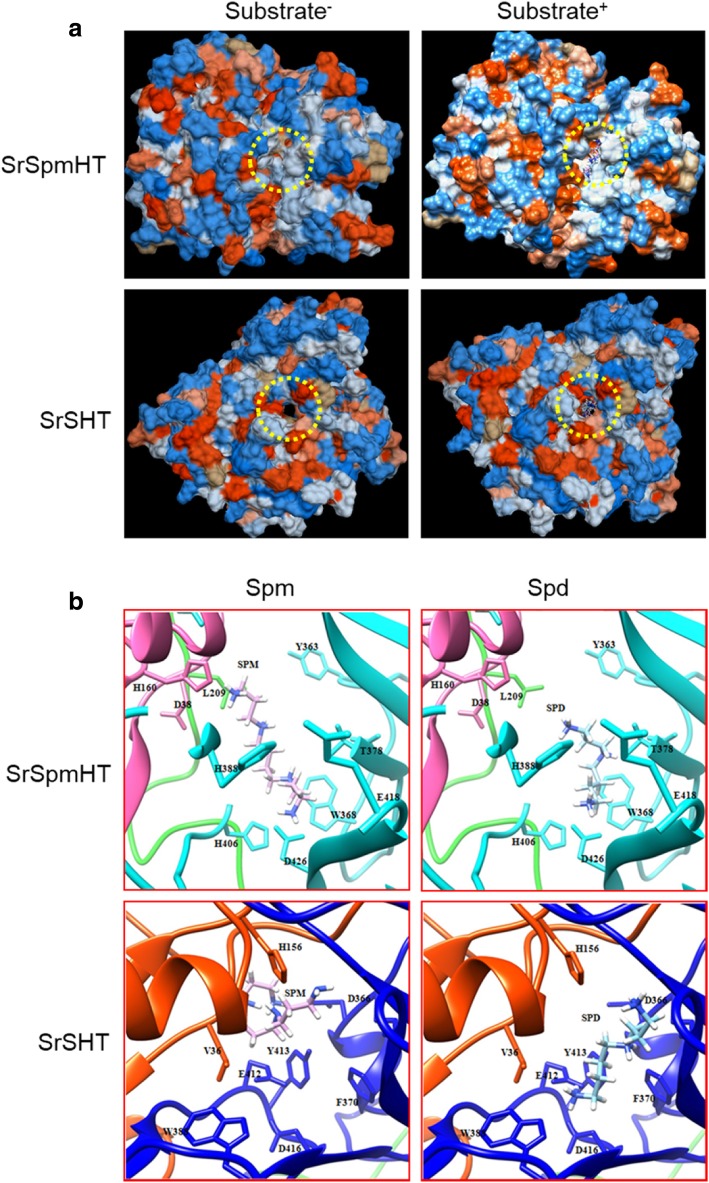
Spatial interaction of spermine/spermidine and SrSpmHT/SrSHT. **a**, The 3D structure of SrSpmHT and SrSHT. Substrate binding pocket was marked with dotted yellow cycle. **b**, Comparison of the best docking results of spermidine and spermine in SrSHT or SrSpmHT active site. SrSHT or SrSpmHT structure is depicted in ribbon representation. For SrSHT, the N-terminal domain (residues 1–191) is colored in orange red and the C-terminal domain (residues 227–449) is colored in blue. The conserved and catalytic residues His156 is shown in side chain/base. The large crossover loop (amino acids 191-227) that connects both domains is marked in green. For SrSpmSHT, the N-terminal domain (residues 6–199) is colored in hot pink and the C-terminal domain (residues 223–447) is colored in cyan. The conserved and catalytic residues His160 is shown in side chain/base. The large crossover loop (amino acids 199-223) that connects both domains is marked in green. The spermidine is represented as a blue and stick model, and the spermine is represented as a pink and stick model. Visualization is performed using UCSF Chimera

We further examined the expression of the spermidine synthase gene (*SpdS*) and spermine synthase gene (*SpmS*) in tissues of both species as indicators of whether plant tissues contained the respective polyamines. As shown in Fig. 6, *SpdS* was expressed in all tissues in both species although at higher levels in eggplant leaves and flowers. In contrast, the expression of *SpmS* in *S. richardii* was significantly higher than that in cultivated eggplant in all examined tissues (Fig. 5); *SpmS* transcript was barely detectable in eggplant fruit. The highest expression of *SpmS* was detected in young fruit of *S. richardii*. These results suggest that spermidine is available in both species, but spermine is mainly synthesized in *S. richardii*. Altogether, these results suggest that spermine is available for SpmHT to form HCSpm *in planta*.

### Structurally SpmHT prefers spermine as the acyl acceptor substrate

Similarity models of SrSpmHT as well as SrSHT were constructed based on the crystal structures of a *Rauvolfia serpentina* vinorine synthase (RsVS) and a coffee shikimate HT (CcHCT), respectively, because among seven structurally known plant BAHD proteins RsVS and CcHCT show the highest identity (27.3 and 37.0%) to SrSpmHT and SrSHT, respectively [31, 46]. The predicted structure of SrSpmHT superimposed well on that of RsVS, as did the predicted structure of SrSHT on that of CcHCT (Additional file 5: Figure S5). Similar to other BAHD family members, tertiary structures of both SrSpmHT and SrSHT were composed of two nearly equal-sized domains consisting of a large mixed β-sheet flanked by α-helices (Additional file 6: Figure S6). This observation indicated that the structures of BAHD family proteins are highly conserved though their sequences have low similarity. It has been reported that the putative reaction center is a central solvent channel in BAHD family proteins [31, 46]. Interestingly, the channel running through the entire SrSHT is clearly a hole, whereas that of SrSpmHT is not (Fig. 6a).

To examine the interaction of SrSHT and SrSpmHT with polyamines, we performed a docking analysis of Spd or Spm in the SrSHT or SrSpmHT active site. For SrSHT, Spd and Spm extended through the solvent channel into the pocket formed by conserved residues Val-36, Asp-366, Phe-370, Trp-388, Glu-412, Tyr-413 and Asp-416. Spd showed Full Fitness of − 2469.339 kcal/mol and ΔG of − 7.936 kcal/mol for the most favorable interaction, whereas Spm had Full Fitness of − 2420.925 kcal/mol and estimated ΔG of − 6.946 kcal/mol. Spd and Spm formed hydrogen-bonding interactions through Glu412, and Asp366, respectively. The amino group of both Spd and Spm extended toward the catalytic site His156, but the amino group of Spd was a better fit because it was straight while Spm formed a ring (Fig. 6b). For SrSpmHT, Spd and Spm extended through the solvent channel into the pocket formed by conserved residues Asp-38, Leu-209, Tyr-363, Trp-368, His-388, His-406, Glu-418 and Asp-426. Spm showed Full Fitness of − 2759.90 kcal/mol and ΔG of − 8.888 kcal/mol for the most favorable interaction, whereas Spd showed Full Fitness of − 2721.611 kcal/mol and estimated ΔG of − 8.649 kcal/mol. Spd and Spm extended through the solvent channel toward the catalytic site H160 from the back face and formed hydrogen-bonding interactions through the highly conserved residues His-406 and Asp-426, and both were stable in the pocket. However, Spm was nearer to catalytic site His160 than Spd (Fig. 6b). These results suggest that structurally SpmHT prefers Spm rather than Spd, whereas SHT prefers Spd to Spm.

## Discussion

Like most solanaceous plants that have been phytochemically analyzed, *Solanum* fruit crops such as eggplant are rich in HCAAs with spermidine or putrescine, but not spermine, as the polyamine moiety [33, 38]. However, HCSpms possess medicinal properties distinct from those of the other, much more common HCAAs [14–18]. Although HCSpms have been known for a few decades as rare plant HCAAs of potential therapeutic value, the committed enzyme catalyzing the condensation of hydroxycinnamoyl-CoA with spermine has not been identified so far [47]. In this study, we identified and characterized SpmHT, a spermine exclusive HT from *S. richardii*, a wild eggplant relative found in Africa.

Structurally SpmHT shares linear similarities to other polyamine hydroxycinnamoyl transferase family enzymes, such as spermidine HT (SHT) and putrescine HT (PutHT). However, SpmHT has two unique features compared with other HTs. First, SpmHT has the highest activity among all known polyamine HTs, as its *V*max is more than 5, 7, 14, 21, and 84 times that of SbHCT, SrSHT, AtSDT, AtSCT, and CcHCT, respectively [2, 26, 31, 32]. Second, SpmHT only utilizes spermine as acyl acceptor. Several catalytic-activity-related residues have been identified in HTs by mutagenesis, such as Thr-36 and Ser-38 in SbHCT. These two residues are specifically involved in hydroxycinnamoyl moiety binding [31, 32]. The positions corresponding to Thr-36 and Ser-38 are replaced by Leu in SpmHTs, and by Val and Thr in SHTs (Additional file 1: Figure S1). Whether these two substitutions in SpmHT have an impact on the catalytic activity is worth further study. As far as the polyamine moiety binding, molecular docking indicates that SpmHT prefers spermine to spermidine based on FullFitness and cluster formation. Three residues (His-406, Asp-426, and Glu-428) involved in the formation of hydrogen bonds are highly conserved in all putative SpmHTs, whereas the corresponding sites in SrSHT are substituted by distinct residues in SrSHT. Further structural study is needed to address how SpmHT specifically selects the acceptor substrate and why it has high enzyme activity.

Our previous analyses show that HCSpms in *S. richardii* fruit are mainly di- or tri- hydrocaffeoyl acylated spermine [19, 33, 39, 40]. However in vitro studies exhibited a specific monohydrocaffeoyl acylation of spermine by SpmHT. It is not clear whether SpmHT can use di- or tri- hydrocaffeoyl CoA as the substrate *in planta*. Another possibility is that monosubstituted spermine conjugate may be an acyl acceptor for a second hydroxycinnamoyl transfer by another acyltransferase [2]. In the case of two native tobacco HTs (NaDH29 and NaCV86), NaDH29 mediates the initial acylation step specifically on Spd and is not able to perform the second acylation. Further acylation is committed by NaCV86 to act on monoacylated spermidines [48]. Further elucidation requires structural or mutagenesis studies and in vivo functional analysis.

SpmHT is highly expressed in fruit of *S. richardii* but barely in any tissue of *S. melongena* or other relatives we profiled [49]. Therefore, SpmHT has been selectively silenced in eggplant and many other plants, suggesting that the reduced expression of SpmHT in eggplant fruit would represent an interesting model to investigate molecular mechanism of eggplant phytochemical evolution during the domestication process. High expression of *SpmS* ensures the sufficiency of Spm for HCSpm synthesis in *S. richardii* fruit. In contrast, there is almost no expression of *SpmS* in *S. melongena* fruits. Hence HCAA composition in *S. richardii* might be achieved through coordinated expression of HCAA structural genes *SpmHT* and *SpmS*. Their coordinated expression may be regulated by some master regulators. MYBs have been shown in other species to regulate *HCT* expression. For example, in *Nicotiana attenuata*, MYB8 controls phenolamide (e.g. HCAA) levels by directly activating the transcription of three polyamine hydroxycinnamic acid transferases (HTs) [25]. Since then, they have been similarly implicated in both monocots and eudicots such as strawberry [49]. A recent study showed that a positive regulator ORA59 could bind to the promoter of an Arabidopsis agmatine coumaroyl transferase (AtACT) and enabled its expression and HCAAs biosynthesis to respond to simultaneous activation of the JA/ET signaling pathways [50]. It would be interesting to identify the master regulators involved in SpmHT biosynthesis in order to activate the pathway and study the function of SpmHT.

High levels of *SpmS* expression suggest that abundant Spm is synthesized in *S. richardii* fruit. This is unusual because Spm is synthesized at lower levels than Spd in most plants [51, 52]. Generally speaking, Spd is thought to contribute to higher vegetative growth, less shriveling (in tomato fruit), and longer life span in transgenic plants overexpressing *SpdS* [52, 53], while elevated Spm promotes abiotic (osmotic and/or salt) and biotic (cucumber mosaic virus and *Pseudomonas viridiflava*) stress tolerance by inducing the expression of defense genes in plants [54–60]. Hence, it appears that Spd is largely implicated in developmental processes, whereas Spm is more likely involved in stress response [53, 61–65]. However, excessive Spm may cause abnormal development [66] and consequently there must be tight regulation of polyamine homeostasis for normal growth of plants [67].

## Conclusions

SrSpmHT is a novel, highly efficient spermine hydroxycinnamoyl transferase that uses Spm exclusively as the acyl acceptor substrate to produce HCSpms. This gene is almost silenced in eggplant and many other plants. This foundational work in *Solanum* should foster broader characterization of SpmHT across the plant kingdom, as well as promote functional analysis of other genes in the phenylpropanoid pathway, which will advance our understanding of the regulation and mechanisms of the HCAA biosynthetic pathway in plants. Unique substrate specificity and high activity of SrSpmHT will facilitate structure-function study of the catalytic mechanism of HT. Further, SrSpmHT could be used to produce HCSpm in bioreactor systems, and the *SrSpmHT* gene used to engineer HCAA metabolism to improve stress tolerance and/or nutritional quality in agricultural produce. Perhaps this could most readily be achieved in eggplant, which is already equipped with the functional gene *SmSpmHT*.

## Methods

### Plant materials

The seeds of eggplant wild relative *S. richardii* (Collection ID: PI500922) were obtained from USDA Germplasm Resource Information Network. The seeds of four *S. melongena* cultivars (Black Beauty, Charming, Santana, and Prosperosa) were purchased from online store eCRATER (https://www.ecrater.com/c/70/home-garden). Plants were grown at Beltsville, MD, as described in a previous report [26]. Roots, leaves, flowers, young fruits at 10 days post anthesis (DPA), and market harvest fruits (20-25 DPA) were collected and frozen in liquid nitrogen and stored at − 80 °C for future use. Three biological replicates of tissues were collected per accession.

### Phytochemical analysis

Sample preparation and HPLC analysis were performed as previously described [40], using a methanol:water solvent. Preliminary identification of major constituents was based on a comparison of HPLC elution times and absorbance spectra (200–650 nm) with those of authentic standards obtained from Dr. Jeffrey Atkinson’ s group at Brock University in Canada [18]. Further identification of HCAA conjugates was done with HR-ESI-MS and ^1^H-NMR as previously described [40].

### In silico analysis and molecular docking

The coding sequences (CDSs) of Arabidopsis spermidine hydroxycinnamoyl transferase (*AtSHT*) was used to blast against eggplant EST database in NCBI, and assembled into contigs. In addition, sequences of 33 BAHD and BAHD-like genes were collected from GenBank or genome databases (e.g., Sol Genomics Network; https://solgenomics.net) of specific plants through BLASTp. Multiple sequence alignment was performed using ClustalW and a gene tree was constructed using Neighbor-Joining (NJ) with 1000 bootstrap replicates in Geneious Pro (4.8.5). To obtain the eggplant spermidine synthase (*SmSPDS*) and spermine synthase (*SmSPMS*), the coding sequences (CDSs) of Arabidopsis *AtSPDS* and spermine synthase *AtSPMS* were used to BLAST against the eggplant genome [68].

For 3D structural analysis, the models of *S. richardii* SHT (SrSHT) and SrSpmHT were constructed using the Swiss-model (http://swissmodel.expasy.org) based on the crystal structure of coffee HT (CcHCT, pdb code: 4G0B) [31] and *Rauvolfia serpentina* HT (RsVS, pdb code: 2BGH), respectively. The model and the template were compared using the Swiss PDB viewer (http://Spdbv.vital-it.ch) [69].

For molecular docking, the computation of the interaction of *S. richardii* SrSHT and SrSpmHT with polyamines (spermidine and spermine) was performed using SwissDock [70]. Full Fitness and Gibbsfree energy (ΔG) of each run (300 runs) of the docking were evaluated. Favorable binding modes were scored based on Full Fitness and cluster formation. Ranking of the cluster was performed using the value of Full Fitness. Results obtained from the SwissDock were visualized by UCSF Chimera (https://www.cgl.ucsf.edu/chimera/).

### Gene expression analysis

RNA isolation, cDNA synthesis, and quantitative PCR were performed as described in previous publication [26]. Gene specific primers for quantitative real-time PCR (qPCR) were designed using Primer3 [71] to amply *SpmHT* and other putative HT, as well as spermine synthesis (*SpmS*), spermidine synthesis (*SpdS*), and *SmGAPDH* (JX448343; an inner control). Gene specific primers are listed in Additional file 7: Table S1. The following thermal cycle was used on CFX96 Touch™ Real-Time PCR Detection System (*BIO-RAD*, Hercules, CA, USA): 95 °C for 2 min and 45 cycles of 95 °C for 5 s, 60 °C for 15 s, followed by a melting curve analysis. Relative quantification of specific mRNA levels was analyzed using the cycle threshold (Ct) 2^−ΔΔCt^ method. Relative expression levels were normalized using the expression of *GAPDH* and shown in fold changes (lowest value = 1). Tukey HSD test was used to determine the significant difference of relative expression of individual genes among different tissues. Experiments were repeated three times (three biological replicates).

### Cloning of *SpmHT* genes

The full-length ORF of *SmSpmHT* was amplified from mature fruit cDNA of *S. melogena* (cv. Black Beauty) using the high fidelity Pfx DNA Polymerase (Invitrogen, Frederick, MD, USA) and the gene-specific primer pair (ATGAAAGATTCGATGCAAGTAAA/CTAAAATTTAGCAAAATCCATGATA). The degenerate primer pair (ATGAAAGATTCGATGCAAGTDAADAT/AAATTTAGCAAAATCSATGATATCYTG) was used to amplify the full length of *SrSpmHT* gene from mature fruit cDNA of *S. richardii*. PCR products were ligated into pCR®4-TOPO® (Invitrogen, Frederick, MD, USA) and verified by Sanger DNA sequencing (Iowa State University, Ames, Iowa, USA).

### Expression and purification of recombinant SpmHT proteins

Both *S. melongena* and *S. richardii* SpmHT CDSs were inserted into the pRham™ N-His SUMO Kan vector (Lucigen, Middleton, WI, USA) in frame with an N-terminal His tag following the manufacturer’s instructions. Briefly, a single colony carrying different constructs was cultured in LB medium containing 50 μg/mL kanamycin in an incubator shaking at 230 rpm at 37 °C. When the OD_600_ value of the culture reached 0.5, the inducer rhamnose was added into the culture at a final concentration of 0.2%. The culture was continually shaken at 37 °C for 5 h. His-tagged proteins were purified under native conditions and SUMO tags were removed using SUMO Express Protease. Concentration of collected proteins was determined by Bradford assay [72] and the purity of recombinant protein was confirmed by SDS-PAGE and Western Blot analysis using the anti-His antibody (Millipore, Gibbstown, NJ, USA). Aliquots of purified proteins were stored at − 80 °C.

### Enzyme activity assay

Hydroxycinnamoyl-CoAs of *p*-coumaric, caffeic and ferulic acids were synthesized using eggplant recombinant 4CL enzyme as described [26]. Enzyme activity of recombinant SpmHT was examined as previously described [26]. The standard reaction was run with 120 μM acyl donor (hydroxycinnamoyl-CoA) and 2.5 mM acyl acceptor (polyamine) in 0.1 M Tris-HCl buffer containing 10 mM EDTA at pH 9.0. The reaction was stopped by adding one volume of 0.4% phosphoric acid after incubating at 30 °C for 15 min. The microplates were read at 358 nm, 354 nm and 342 nm for monitoring caffeoyl-CoA, feruloyl-CoA, and ρ-coumaroyl-CoA, respectively, with EON microplate reader (BioTek, Winooski, VT, USA). Blank controls contained all components except for polyamine. The kinetic constants for SrSpmHT were determined using 0 to 240 μM different hydroxycinnamoyl-CoAs at 2.5 mM of acyl acceptor substrate (spermine). pH optima were determined by performing the assay in 0.1 M Tris-HCl buffered to different pHs in the range 7.0 to 10.0. All the reactions were run in duplicate, and each experiment was repeated at least twice.

### Enzyme kinetic analysis

Kinetics data were fitted to the Michaelis-Menten equation. The turnover number *K*_cat_ equals *V*_max_/[E], in which [E] is the enzyme mole concentration. *K*_cat_/*K*_m_ is used to describe the catalytic efficiency of an enzyme. All data were fit with KaleidoGraph version 4.5 from Synergy Software. The kinetic parameters were derived from at least three determinations. Multiple comparison of mean values of kinetic parameters was done with Tukey HSD test after one-way ANOVA statistical analysis.

## Additional files

Additional file 1: **Figure S1.** Amino acid sequence alignment of BAHD-like proteins. (PDF 994 kb)

Additional file 2: **Figure S2.** Enzymological analysis of recombinant SrSpmHT. (PDF 409 kb)

Additional file 3: **Figure S3.** SrSpmHT catalytic kinetics toward hydroxycinnamoyl CoA. (PDF 261 kb)

Additional file 4: **Figure S4.** Isolation and characterization of recombinant *S. melongena* SpmHT. (PDF 214 kb)

Additional file 5: **Figure S5.** Structural alignment of predicted SrSpmHT, SrSHT and their modeling templates. (PDF 464 kb)

Additional file 6: **Figure S6.** Spatial structure of SrSpmHT and SrSHT. (PDF 306 kb)

Additional file 7: **Table S1.** List of primers for quantitative PCR analysis. (PDF 287 kb)

## Data Availability

Genbank accession numbers of SrSpmHT and SmSpmHT are KR150683 and KP233218 respectively**.** All the data supporting our findings are contained within the manuscript. Constructs and seeds are available from TY on reasonable request.

## Abbreviations

HCAA: Hydroxycinnamic acid amides
HCSpd: Hydroxycinnamoyl spermidine
HCSpm: Hydroxycinnamoyl spermine
HT: Hydroxycinnamoyl transferase
SpdHT: Spermidine hydroxycinnamoyl transferase
SpmHT: Spermine hydroxycinnamoyl transferase

## References

[1] MartinTanguy J (1997). Conjugated polyamines and reproductive development: biochemical, molecular and physiological approaches. Physiol Plant.

[2] Luo J, Fuell C, Parr A, Hill L, Bailey P, Elliott K, Fairhurst SA, Martin C, Michael AJ (2009). A novel polyamine acyltransferase responsible for the accumulation of spermidine conjugates in Arabidopsis seed. Plant Cell.

[3] Funayama S, Yoshida K, Konno C, Hikino H (1980). Structure of kukoamine-a, a hypotensive principle of lycium-Chinense root barks. Tetrahedron Lett.

[4] Bouchereau A, Aziz A, Larher F, Martin-Tanguy J (1999). Polyamines and environmental challenges: recent development. Plant Sci.

[5] Bassard JE, Ullmann P, Bernier F, Werck-Reichhart D (2010). Phenolamides: bridging polyamines to the phenolic metabolism. Phytochemistry.

[6] Kwon YI, Apostolidis E, Shetty K (2008). *In vitro* studies of eggplant (*Solanum melongena*) phenolics as inhibitors of key enzymes relevant for type 2 diabetes and hypertension. Bioresour Technol.

[7] Shahidi F, Chandrasekara A (2010). Hydroxycinnamates and their *in vitro* and *in vivo* antioxidant activities. Phytochem Rev.

[8] Ma CH, Dastmalchi K, Whitaker BD, Kennelly EJ (2011). Two new antioxidant malonated caffeoylquinic acid isomers in fruits of wild eggplant relatives. J Agric Food Chem.

[9] Parr AJ, Mellon FA, Colquhoun IJ, Davies HV (2005). Dihydrocaffeoyl polyamines (kukoamine and allies) in potato (*Solanum tuberosum*) tubers detected during metabolite profiling. J Agric Food Chem.

[10] Dong XK, Gao YQ, Chen W, Wang WS, Gong L, Liu XQ, Luo J (2015). Spatiotemporal distribution of phenolamides and the genetics of natural variation of hydroxycinnamoyl spermidine in rice. Mol Plant.

[11] Martintanguy J (1985). The occurrence and possible function of hydroxycinnamoyl acid-amides in plants. Plant Growth Regul.

[12] Ponasik JA, Strickland C, Faerman C, Savvides S, Karplus PA, Ganem B (1995). Kukoamine-a and other hydrophobic acylpolyamines - potent and selective inhibitors of crithidia-fasciculata trypanothione reductase. Biochem J.

[13] Krieger S, Schwarz W, Ariyanayagam MR, Fairlamb AH, Krauth-Siegel RL, Clayton C (2000). Trypanosomes lacking trypanothione reductase are avirulent and show increased sensitivity to oxidative stress. Mol Microbiol.

[14] Fairlamb AH, Cerami A (1992). Metabolism and functions of trypanothione in the kinetoplastida. Annu Rev Microbiol.

[15] Tovar J, Wilkinson S, Mottram JC, Fairlamb AH (1998). Evidence that trypanothione reductase is an essential enzyme in Leishmania by targeted replacement of the tryA gene locus. Mol Microbiol.

[16] Wang QP, Li HY, Sun Z, Dong LH, Gao L, Liu CL, Wang XJ (2016). Kukoamine a inhibits human glioblastoma cell growth and migration through apoptosis induction and epithelial-mesenchymal transition attenuation. Sci Rep.

[17] Li GY, Zhou F, Chen Y, Zhang WG, Wang N (2017). Kukoamine a attenuates insulin resistance and fatty liver through downregulation of Srebp-1c. Biomed Pharmacother.

[18] Fixon-Owoo S, Levasseur F, Williams K, Sabado TN, Lowe M, Klose M, Joffre Mercier A, Fields P, Atkinson J (2003). Preparation and biological assessment of hydroxycinnamic acid amides of polyamines. Phytochemistry.

[19] Vogt T (2010). Phenylpropanoid biosynthesis. Mol Plant.

[20] Hu Y, Gai Y, Yin L, Wang X, Feng C, Feng L, Li D, Jiang XN, Wang DC (2010). Crystal structures of a Populus tomentosa 4-coumarate:CoA ligase shed light on its enzymatic mechanisms. Plant Cell.

[21] Hedberg C, Hesse M, Werner C (1996). Spermine and spermidine hydroxycinnamoyl transferases in *Aphelandra tetragona*. Plant Sci.

[22] Wiermann R, Gubatz S (1992). Pollen wall and sporopollenin. Int Rev Cytol.

[23] Meurer B, Wiermann R, Strack D (1988). Phenylpropanoid patterns in fagales pollen and their phylogenetic relevance. Phytochemistry.

[24] Grienenberger E, Besseau S, Geoffroy P, Debayle D, Heintz D, Lapierre C, Pollet B, Heitz T, Legrand M (2009). A BAHD acyltransferase is expressed in the tapetum of Arabidopsis anthers and is involved in the synthesis of hydroxycinnamoyl spermidines. Plant J.

[25] Onkokesung N, Gaquerel E, Kotkar H, Kaur H, Baldwin IT, Galis I (2012). MYB8 controls inducible phenolamide levels by activating three novel hydroxycinnamoyl-coenzyme a: polyamine transferases in *nicotiana attenuata*. Plant Physiol.

[26] Peng H, Yang T, Whitaker BD, Trouth F, Shangguan L, Dong W, Jurick WM (2016). Characterization of spermidine hydroxycinnamoyl transferases from eggplant (*Solanum melongena* L.) and its wild relative *Solanum richardii* Dunal. Hortic Res.

[27] St-Pierre B, De Luca V (2000). Evolution of acyltransferase genes: origin and diversification of the BAHD superfamily of acyltransferases involved in secondary metabolism. Recent Adv Phytochem.

[28] D'Auria JC (2006). Acyltransferases in plants: a good time to be BAHD. Curr Opin Plant Biol.

[29] Carqueijeiro I, Duge de Bernonville T, Lanoue A, Dang TT, Teijaro CN, Paetz C, Billet K, Mosquera A, Oudin A, Besseau S (2018). A BAHD acyltransferase catalyzing 19-O-acetylation of tabersonine derivatives in roots of Catharanthus roseus enables combinatorial synthesis of monoterpene indole alkaloids. Plant J.

[30] Yu XH, Chen MH, Liu CJ (2008). Nucleocytoplasmic-localized acyltransferases catalyze the malonylation of 7-O-glycosidic (iso)flavones in *Medicago truncatula*. Plant J.

[31] Lallemand LA, Zubieta C, Lee SG, Wang YC, Acajjaoui S, Timmins J, McSweeney S, Jez JM, McCarthy JG, McCarthy AA (2012). A structural basis for the biosynthesis of the major chlorogenic acids found in coffee. Plant Physiol.

[32] Walker AM, Hayes RP, Youn B, Vermerris W, Sattler SE, Kang C (2013). Elucidation of the structure and reaction mechanism of sorghum hydroxycinnamoyltransferase and its structural relationship to other coenzyme A-dependent transferases and synthases. Plant Physiol.

[33] Wu SB, Meyer RS, Whitaker BD, Litt A, Kennelly EJ (2013). A new liquid chromatography-mass spectrometry-based strategy to integrate chemistry, morphology, and evolution of eggplant (*Solanum*) species. J Chromatogr A.

[34] Weese TL, Bohs L (2010). Eggplant origins: out of Africa, into the orient. Taxon.

[35] Wu SJ, Ng LT, Lin CC (2004). Antioxidant activities of some common ingredients of traditional Chinese medicine, *Angelica sinensis*, *Lycium barbarum* and *Poria cocos*. Phytother Res.

[36] Meyer RS, Bamshad M, Fuller DQ, Litt A (2014). Comparing medicinal uses of eggplant and related solanaceae in China, India, and the Philippines suggests the independent development of uses, cultural diffusion, and recent species substitutions. Econ Bot.

[37] Hanson PM, Yang RY, Tsou SCS, Ledesma D, Engle L, Lee TC (2006). Diversity in eggplant (*Solanum melongena*) for superoxide scavenging activity, total phenolics, and ascorbic acid. J Food Compos Anal.

[38] Whitaker BD, Stommel JR (2003). Distribution of hydroxycinnamic acid conjugates in fruit of commercial eggplant (*Solanum melongena* L.) cultivars. J Agric Food Chem.

[39] Stommell JR, Whitaker BD (2003). Phenolic acid content and composition of eggplant fruit in a germplasm core subset. J Am Soc Hortic Sci.

[40] Meyer RS, Whitaker BD, Little DP, Wu SB, Kennelly EJ, Long CL, Litt A (2015). Parallel reductions in phenolic constituents resulting from the domestication of eggplant. Phytochemistry.

[41] Kaushik PGP, Vilanova S, Raigón MD, Prohens J, Plazas M (2017). Phenolics content, fruit flesh colour and browning in cultivated eggplant, wild relatives and interspecific hybrids and implications for fruit quality breeding. Food Res Int.

[42] Taher D, Solberg SO, Prohens J, Chou YY, Rakha M, Wu TH (2017). World vegetable center eggplant collection: origin, composition, seed dissemination and utilization in breeding. Front Plant Sci.

[43] Okmen B, Sigva HO, Mutlu S, Doganlar S, Yemenicioglu A, Frary A (2009). Total antioxidant activity and total phenolic contents in different Turkish eggplant (*solanum melongena* L.) cultivars. Int J Food Prop.

[44] Sudheesh S, Presannakumar G, Vijayakumar S, Vijayalakshmi NR (1997). Hypolipidemic effect of flavonoids from *Solanum melongena*. Plant Food Hum Nutr.

[45] Meyer RS, Little DP, Whitaker BD, Litt A. The Genetics of Eggplant Nutrition. In: Chapman M, editor. The Eggplant Genome: Springer Verlag; 2019.

[46] Ma XY, Koepke J, Panjikar S, Fritzsch G, Stockigt J (2005). Crystal structure of vinorine synthase, the first representative of the BAHD superfamily. J Biol Chem.

[47] Luo J, Nishiyama Y, Fuell C, Taguchi G, Elliott K, Hill L, Tanaka Y, Kitayama M, Yamazaki M, Bailey P (2007). Convergent evolution in the BAHD family of acyl transferases: identification and characterization of anthocyanin acyl transferases from *Arabidopsis thaliana*. Plant J.

[48] Meyer RS, Karol KG, Little DP, Nee MH, Litt A (2012). Phylogeographic relationships among Asian eggplants and new perspectives on eggplant domestication. Mol Phylogenet Evol.

[49] Liu JY, Osbourn A, Ma PD (2015). MYB transcription factors as regulators of Phenylpropanoid metabolism in plants. Mol Plant.

[50] Li J, Zhang K, Meng Y, Hu J, Ding M, Bian J, Yan M, Han J, Zhou M (2018). Jasmonic acid/ethylene signaling coordinates hydroxycinnamic acid amides biosynthesis through ORA59 transcription factor. Plant J.

[51] Adiga PR, Prasad GL (1985). Biosynthesis and regulation of polyamines in higher-plants. Plant Growth Regul.

[52] Eisenberg T, Knauer H, Schauer A, Buttner S, Ruckenstuhl C, Carmona-Gutierrez D, Ring J, Schroeder S, Magnes C, Antonacci L (2009). Induction of autophagy by spermidine promotes longevity. Nat Cell Biol.

[53] Nambeesan S, Datsenka T, Ferruzzi MG, Malladi A, Mattoo AK, Handa AK (2010). Overexpression of yeast spermidine synthase impacts ripening, senescence and decay symptoms in tomato. Plant J.

[54] Sreenivasulu N, Grimm B, Wobus U, Weschke W (2000). Differential response of antioxidant compounds to salinity stress in salt-tolerant and salt-sensitive seedlings of foxtail millet (*Setaria italica*). Physiol Plant.

[55] Roychoudhury A, Basu S, Sengupta DN (2011). Amelioration of salinity stress by exogenously applied spermidine or spermine in three varieties of indica rice differing in their level of salt tolerance. J Plant Physiol.

[56] Zrig A, Tounekti T, Vadel AM, Ben Mohamed H, Valero D, Serrano M, Chtara C, Khemira H (2011). Possible involvement of polyphenols and polyamines in salt tolerance of almond rootstocks. Plant Physiol Biochem.

[57] Radhakrishnan R, Lee IJ (2013). Ameliorative effects of spermine against osmotic stress through antioxidants and abscisic acid changes in soybean pods and seeds. Acta Physiol Plant.

[58] Duan JJ, Li J, Guo SR, Kang YY (2008). Exogenous spermidine affects polyamine metabolism in salinity-stressed Cucumis sativus roots and enhances short-term salinity tolerance. J Plant Physiol.

[59] Mitsuya Y, Takahashi Y, Berberich T, Miyazaki A, Matsumura H, Takahashi H, Terauchi R, Kusano T (2009). Spermine signaling plays a significant role in the defense response of *Arabidopsis thaliana* to cucumber mosaic virus. J Plant Physiol.

[60] Gonzalez ME, Marco F, Minguet EG, Carrasco-Sorli P, Blazquez MA, Carbonell J, Ruiz OA, Pieckenstain FL (2011). Perturbation of spermine synthase gene expression and transcript profiling provide new insights on the role of the tetraamine spermine in Arabidopsis defense against *Pseudomonas viridiflava*. Plant Physiol.

[61] Slocum RD, Kaursawhney R, Galston AW (1984). The physiology and biochemistry of polyamines in plants. Arch Biochem Biophys.

[62] Kaursawhney R, Tiburcio AF, Galston AW (1988). Spermidine and flower-bud differentiation in thin-layer explants of tobacco. Planta.

[63] Imai A, Matsuyama T, Hanzawa Y, Akiyama T, Tamaoki M, Saji H, Shirano Y, Kato T, Hayashi H, Shibata D (2004). Spermidine synthase genes are essential for survival of Arabidopsis. Plant Physiol.

[64] Yamaguchi K, Takahashi Y, Berberich T, Imai A, Takahashi T, Michael AJ, Kusano T (2007). A protective role for the polyamine spermine against drought stress in Arabidopsis. Biochem Biophys Res Commun.

[65] Yamaguchi K, Takahashi Y, Berberich T, Imai A, Miyazaki A, Takahashi T, Michael A, Kusano T (2006). The polyamine spermine protects against high salt stress in *Arabidopsis thaliana*. FEBS Lett.

[66] Takahashi Y, Berberich T, Miyazaki A, Seo S, Ohashi Y, Kusano T (2003). Spermine signalling in tobacco: activation of mitogen-activated protein kinases by spermine is mediated through mitochondrial dysfunction. Plant J.

[67] Tiburcio AF, Altabella T, Bitrian M, Alcazar R (2014). The roles of polyamines during the lifespan of plants: from development to stress. Planta.

[68] Hirakawa H, Shirasawa K, Miyatake K, Nunome T, Negoro S, Ohyama A, Yamaguchi H, Sato S, Isobe S, Tabata S (2014). Draft genome sequence of eggplant (Solanum melongena L.): the representative solanum species indigenous to the old world. DNA Res.

[69] Kiefer F, Arnold K, Kunzli M, Bordoli L, Schwede T (2009). The SWISS-MODEL repository and associated resources. Nucleic Acids Res.

[70] Grosdidier A, Zoete V, Michielin O (2011). SwissDock, a protein-small molecule docking web service based on EADock DSS. Nucleic Acids Res.

[71] Untergasser A, Cutcutache I, Koressaar T, Ye J, Faircloth BC, Remm M, Rozen SG (2012). Primer3-new capabilities and interfaces. Nucleic Acids Res.

[72] Bradford MM (1976). A rapid and sensitive method for the quantitation of microgram quantities of protein utilizing the principle of protein-dye binding. Anal Biochem.

