# Supplementary material for: A novel hydroxycinnamoyl transferase for synthesis of hydroxycinnamoyl spermine conjugates in plants
Source: BMC Plant Biol. 2019 Jun 17;19:261. doi: 10.1186/s12870-019-1846-3 (PMC6580504; doi:10.1186/s12870-019-1846-3)
Supplement: Supplementary file 1 — Figure S1. Amino acid sequence alignment of BAHD-like proteins. (PDF 994 kb) [file 12870_2019_1846_MOESM1_ESM.pdf]

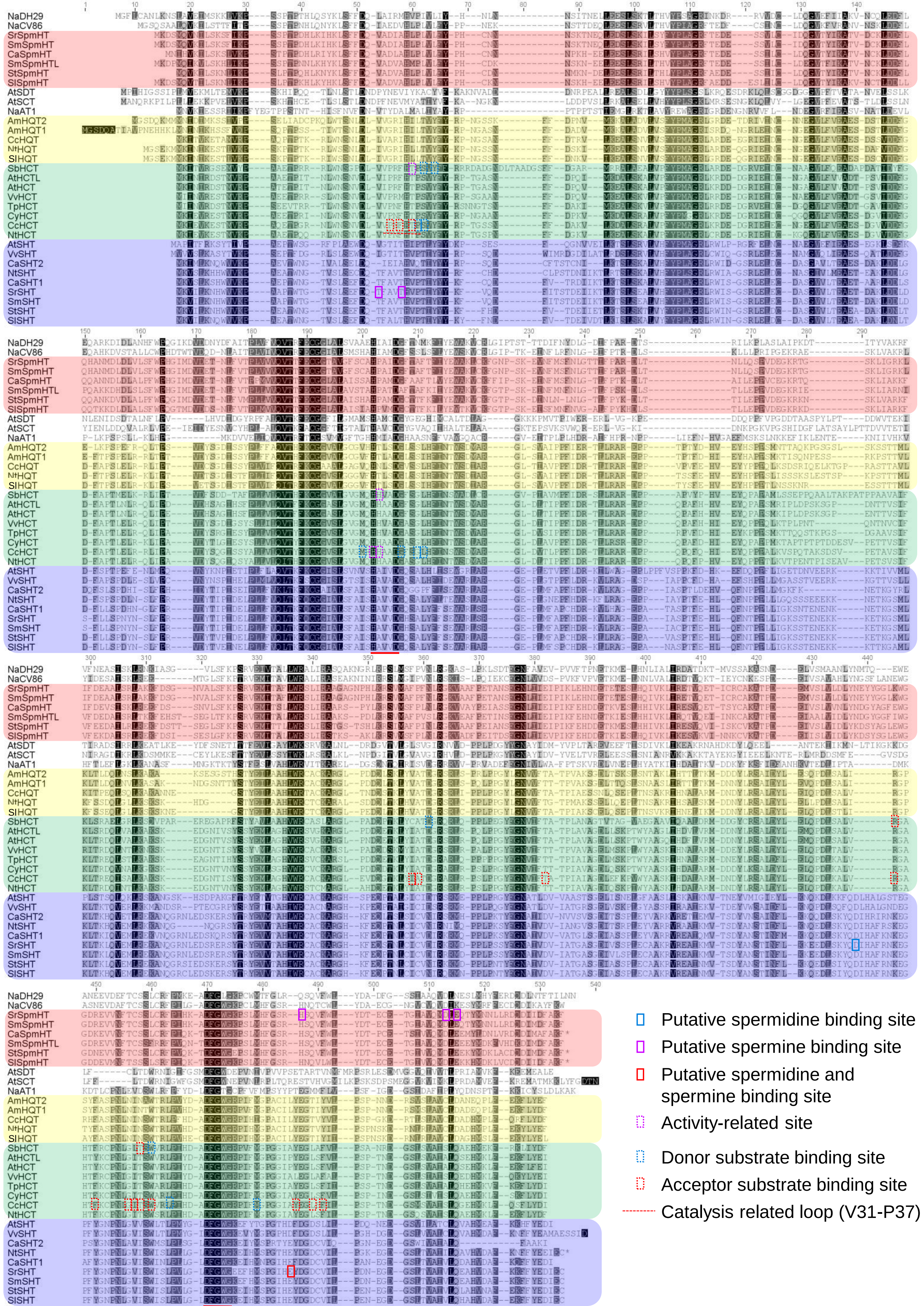
Putative spermidine binding site
Putative spermine binding site
Putative spermidine and spermine binding site
Activity-related site
Donor substrate binding site
Acceptor substrate binding site
Catalysis related loop (V31-P37)

Additional file 1: Figure S1. Sequence alignment of BAHD like proteins. Identical and similar amino acids were shaded in black and grey, respectively. The conserved domains (HXXXD and DFGWG) in BAHD family proteins were underlined with red solid lines. The catalytically important residues were marked with different symbols. Organism resource and accession numbers of included genes are shown in figure 4.
