# Supplementary material for: A novel hydroxycinnamoyl transferase for synthesis of hydroxycinnamoyl spermine conjugates in plants
Source: BMC Plant Biol. 2019 Jun 17;19:261. doi: 10.1186/s12870-019-1846-3 (PMC6580504; doi:10.1186/s12870-019-1846-3)
Supplement: Supplementary file 2 — Figure S2. Enzymological analysis of recombinant SrSpmHT. (PDF 409 kb) [file 12870_2019_1846_MOESM2_ESM.pdf]

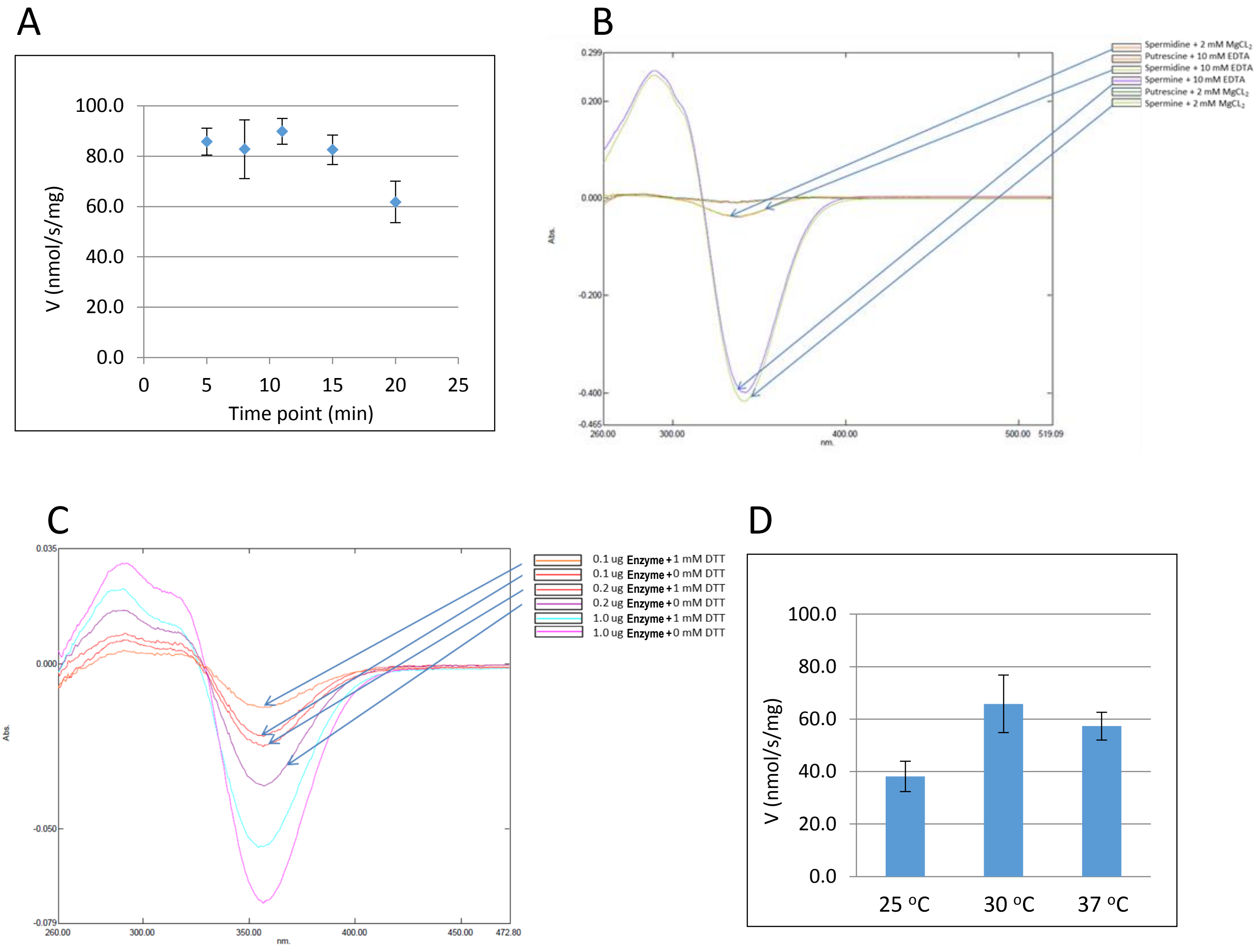


**Additional file 2: Figure S2** Enzymological analysis of recombinant SrSpmHT. A, Initial velocity of SrSpmHT. The activities were measured at 5, 8, 11, 15, and 20 min with 60 µM feruloyl-CoA and 2.5 mM spermine. Bars show standard errors calculated from triplicates. B, Effect of EDTA and $Mg^{2+}$ on activity of SrSpmHT. Reactions were performed with feruloy-CoA and different polyamines (spermidine, spermine, and putrescine) under 10mM EDTA or 2mM $MgCl_2$. The activities were examined according to consumption of donor substrate by checking absorbance (260-520 nm). C, Effect of DTT on activity of SrSpmHT. Reactions were performed with *p*-coumaroyl-CoA and different amount of enzyme under 0 or 1 mM DTT. The activities were examined according to consumption of donor substrate by checking absorbance (260-470 nm). D, Influence of temperature to SrSpmHT activity. Reactions were performed with 60 µM *p*-coumaroyl-CoA and 2.5 mM spermine at different temperature (25 ºC, 30 ºC, and 37 ºC). Bars show standard errors calculated from triplicates.
