# Supplementary material for: A novel hydroxycinnamoyl transferase for synthesis of hydroxycinnamoyl spermine conjugates in plants
Source: BMC Plant Biol. 2019 Jun 17;19:261. doi: 10.1186/s12870-019-1846-3 (PMC6580504; doi:10.1186/s12870-019-1846-3)
Supplement: Supplementary file 3 — Figure S3. SrSpmHT catalytic kinetics toward hydroxycinnamoyl CoA. (PDF 261 kb) [file 12870_2019_1846_MOESM3_ESM.pdf]

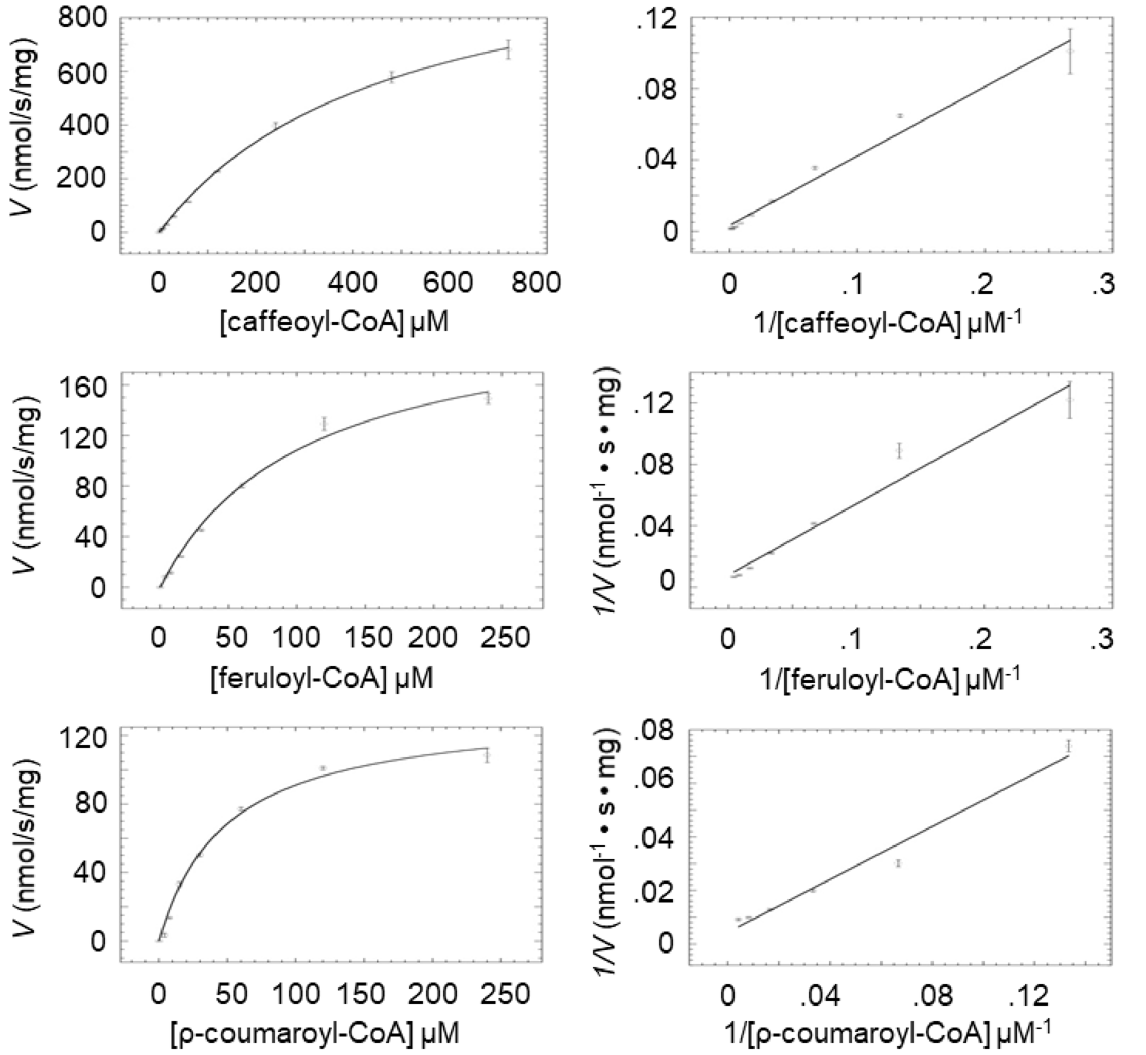

Additional file 3 Figure S3 SrSpmHT catalytic kinetics toward hydroxycinnamoyl CoA. Data were fitted to the Michaelis–Menten equation (left). Each point represents the mean velocity plus SD from triplicate determinations. The Lineweaver-Burk plot (right) is shown to illustrate the kinetics.
