# Supplementary material for: A novel hydroxycinnamoyl transferase for synthesis of hydroxycinnamoyl spermine conjugates in plants
Source: BMC Plant Biol. 2019 Jun 17;19:261. doi: 10.1186/s12870-019-1846-3 (PMC6580504; doi:10.1186/s12870-019-1846-3)
Supplement: Supplementary file 4 — Figure S4. Isolation and characterization of recombinant S. melongena SpmHT. (PDF 214 kb) [file 12870_2019_1846_MOESM4_ESM.pdf]

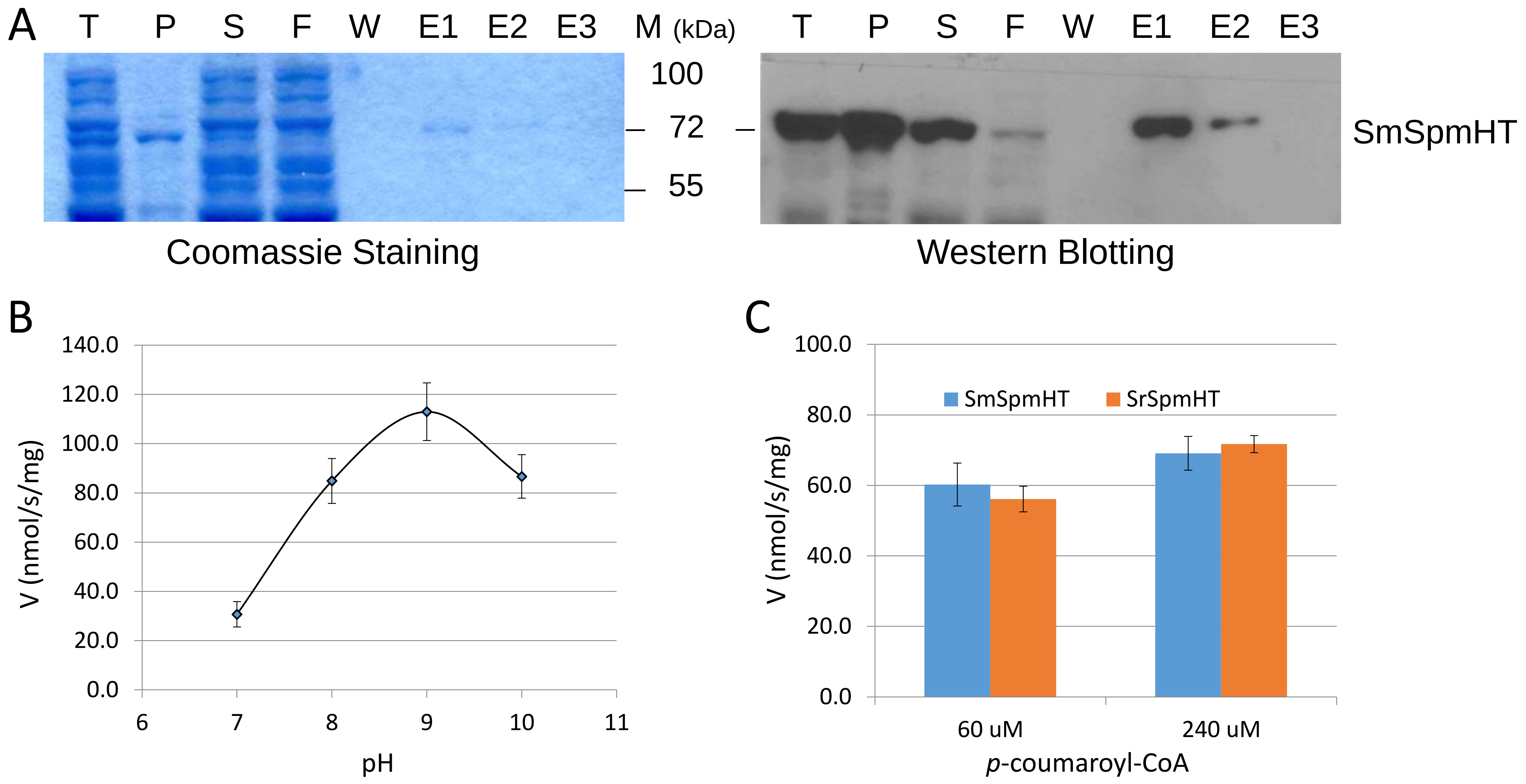

Additional file 4 **Figure S4** Isolation and characterization of recombinant *S. melongena* SpmHT. A), His-tagged recombinant proteins were expressed in *E. coli* and purified from cell lysates by nickel affinity chromatography. Evaluation of protein purity was performed by SDS-PAGE with coomassie blue staining of the gel (left), and Western blotting using anti-His-tag antibody (right): T, total cell lysate; P, cell pellet; S, supernatant; F, Flowthrough solution; W, last column wash; E, column eluates. B), pH-dependent activities of SmSpmHT. Reactions were performed with 60 µM feruloyl-CoA and 2.5 mM spermine at different pH environments. Bars show standard errors calculated from triplicates. C, Reactions were performed with 60 or 240 µM p-coumaroyl-CoA and 2.5 mM spermine with SmSpmHT or SrSpmHT. Bars show standard errors calculated from triplicates.
