# Supplementary material for: A novel hydroxycinnamoyl transferase for synthesis of hydroxycinnamoyl spermine conjugates in plants
Source: BMC Plant Biol. 2019 Jun 17;19:261. doi: 10.1186/s12870-019-1846-3 (PMC6580504; doi:10.1186/s12870-019-1846-3)
Supplement: Supplementary file 5 — Figure S5. Structural alignment of predicted SrSpmHT, SrSHT and their modeling templates. (PDF 464 kb) [file 12870_2019_1846_MOESM5_ESM.pdf]

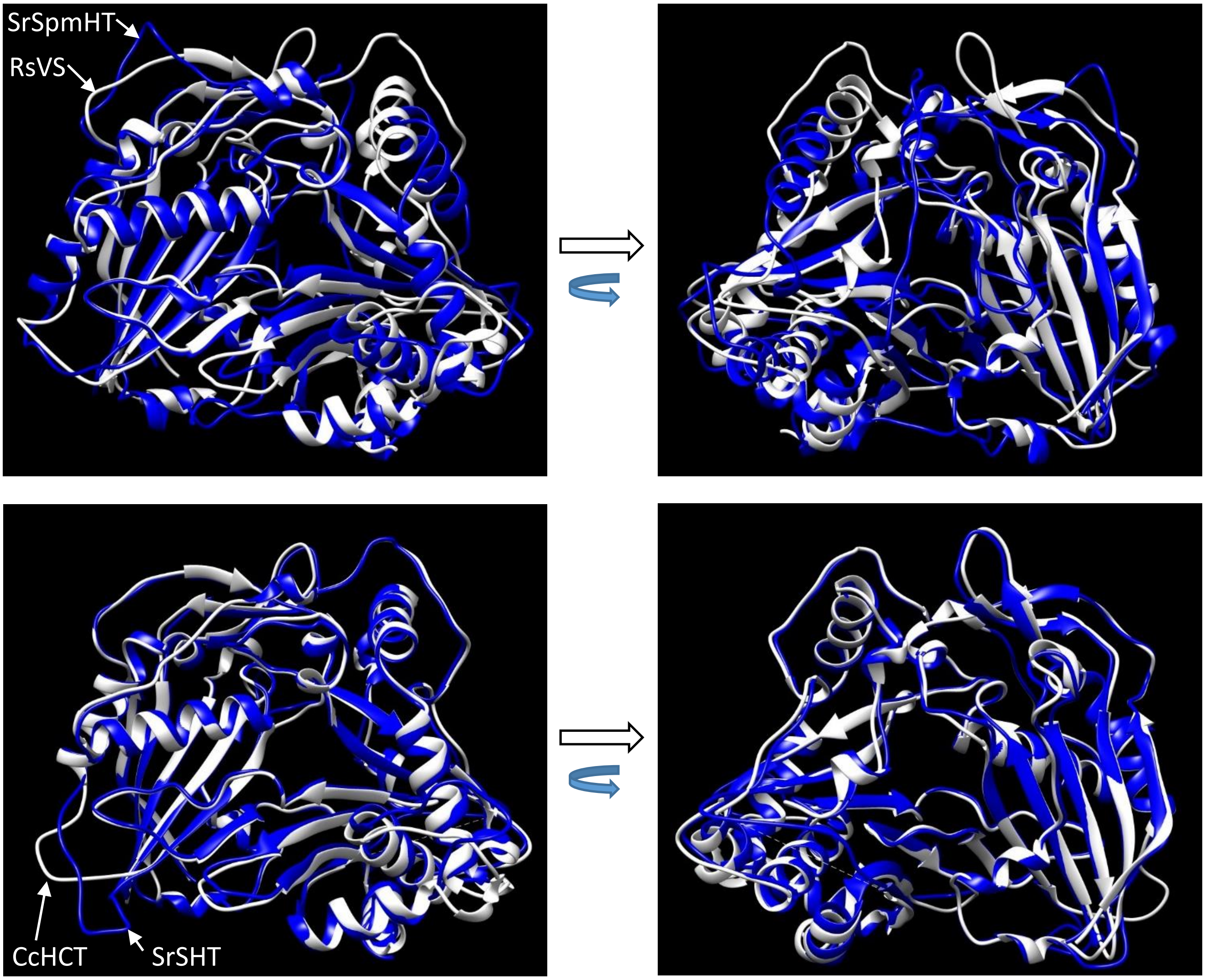

Additional file 5 **Figure S5** Structural alignment of predicted SrSpmHT, SrSHT and their modeling templates (RsVS and CcHCT). Structure modeling for SrSpmHT and SrSHT was performed with Phyre2 online service (http://twitter.com/phyre2server). 3D structures of all proteins were visualized using UCSF Chimera. The image was rotated 180 around a horizontal axis from front view (left) to back view (right).
