# Supplementary material for: A novel hydroxycinnamoyl transferase for synthesis of hydroxycinnamoyl spermine conjugates in plants
Source: BMC Plant Biol. 2019 Jun 17;19:261. doi: 10.1186/s12870-019-1846-3 (PMC6580504; doi:10.1186/s12870-019-1846-3)
Supplement: Supplementary file 6 — Figure S6. Spatial structure of SrSpmHT and SrSHT. (PDF 306 kb) [file 12870_2019_1846_MOESM6_ESM.pdf]

SrSpmHT

SrSHT

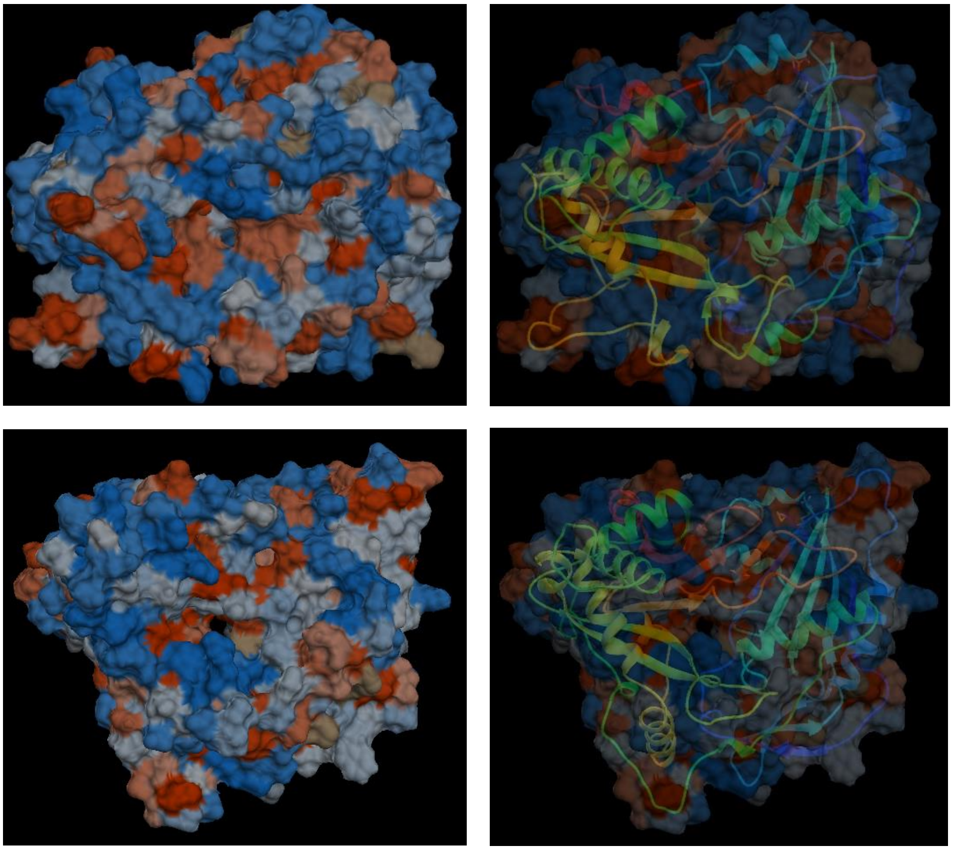

Additional file 6 **Figure S6** Spatial structure of SrSpmHT and SrSHT. Visualization was performed using UCSF Chimera .
