# Supplementary material for: A novel hydroxycinnamoyl transferase for synthesis of hydroxycinnamoyl spermine conjugates in plants
Source: BMC Plant Biol. 2019 Jun 17;19:261. doi: 10.1186/s12870-019-1846-3 (PMC6580504; doi:10.1186/s12870-019-1846-3)
Supplement: Supplementary file 7 — Table S1. List of primers for quantitative PCR analysis. (PDF 287 kb) [file 12870_2019_1846_MOESM7_ESM.pdf]

Additional file 7: Table S1 List of gene specific primers for qPCR

Gene or Locus	Forward/Reverse	Sequence
*SHT1 (SHT)**	F	CGCCAACGTGGACTGGCACTGTTT
	R	CGCAAACGGCCAGCCAATGGATAGA
SHT3	F	TGTGGAGGGCTCTGATCGGTGCTT
	R	CCATGTCCCGGTACAAATTTCACAGGG
SHT4 (SpmHT)	F	CCGCATTATCGAGGCTCGCGAAGA
	R	AAAGCAGCAACCTTGCCGCGTAAGT
SmSPDS	F	TGCCGCCAGATCTTCAAAGGCTCA
	R	GGTCTTGCCATGGCTTTCGTCCTCG
SmSPMS	F	TGTAGGGCCTGCGCTAGAGCTT
	R	GAAGCCACATGCTCTCCGCCAT
SmGAPDH	F	CCGCTCCTAGCAAAGATGCC
	R	ACCCTCCACAATGCCAAACC

Note: * This gene was *SHT* which has been reported [26].
